# The socioeconomic impact of multidrug resistant tuberculosis on patients: results from Ethiopia, Indonesia and Kazakhstan

**DOI:** 10.1186/s12879-016-1802-x

**Published:** 2016-09-05

**Authors:** Susan van den Hof, David Collins, Firdaus Hafidz, Demissew Beyene, Aigul Tursynbayeva, Edine Tiemersma

**Affiliations:** 1 KNCV Tuberculosis Foundation, The Hague, The Netherlands; 2 Amsterdam Institute for Global Health and Development and Academic Medical Center, Amsterdam, The Netherlands; 3 Management Sciences for Health, Medford, MA USA; 4 University of Gadjah Mada, Yogyakarta, Indonesia; 5 Armauer Hansen Research Institute, Addis Ababa, Ethiopia; 6 KNCV Tuberculosis Foundation, Almaty, Kazakhstan

**Keywords:** Tuberculosis, Multi-drug resistance, Patient costs, Cross-sectional survey, Ethiopia, Indonesia, Kazakhstan

## Abstract

**Background:**

One of the main goals of the post-2015 global tuberculosis (TB) strategy is that no families affected by TB face catastrophic costs. We revised an existing TB patient cost measurement tool to specifically also measure multi-drug resistant (MDR) TB patients’ costs and applied it in Ethiopia, Indonesia and Kazakhstan.

**Methods:**

Through structured interviews with TB and MDR-TB patients in different stages of treatment, we collected data on the direct (out of pocket) and indirect (loss of income) costs of patients and their families related to the diagnosis and treatment of TB and MDR-TB. Direct costs included costs for hospitalization, follow-up tests, transport costs for health care visits, and food supplements. Calculation of indirect costs was based on time needed for diagnosis and treatment. Costs were extrapolated over the patient’s total treatment phase.

**Results:**

In total 406 MDR-TB patients and 197 other TB patients were included in the survey: 169 MDR-TB patients and 25 other TB patients in Ethiopia; 143 MDR-TB patients and 118 TB patients in Indonesia; and 94 MDR-TB patients and 54 other TB patients in Kazakhstan. Total costs for diagnosis and current treatment episode for TB patients were estimated to be USD 260 in Ethiopia, USD 169 in Indonesia, and USD 929 in Kazakhstan, compared to USD 1838, USD 2342, and USD 3125 for MDR-TB patients, respectively. These costs represented 0.82–4.6 months of pre-treatment household income for TB patients and 9.3–24.9 months for MDR-TB patients. Importantly, 38–92 % reported income loss and 26–76 % of TB patients lost their jobs due to (MDR) TB illness, further aggravating the financial burden.

**Conclusions:**

The financial burden of MDR-TB is alarming, although all TB patients experienced substantial socioeconomic impact of the disease. If the patient is the breadwinner of the family, the combination of lost income and extra costs is generally catastrophic. Therefore, it should be a priority of the government to relieve the financial burden based on the cost mitigation options identified.

## Background

One of the main goals of the post-2015 global tuberculosis (TB) strategy is that no families affected by TB face catastrophic costs [1]. There is no universal definition of catastrophic costs and a threshold for TB-related catastrophic costs still needs to be defined [2]. Although drugs for TB treatment are free in most high TB-burden countries, TB patients face costs due to charges for related health services, costs for transport, accommodation, nutrition and suffer lost income. A recent systematic review showed that the financial burden of both diagnosis and treatment was high and varied widely across settings, the total costs amounting to 58 % (range 5–306 %) of annual patient income [2]. These costs are expected to be higher for patients with multidrug resistant (MDR) TB than for other TB patients given the three to four times’ longer treatment period. Although there is a paucity of data, the data at hand indicate that, during treatment, patients with MDR-TB face 5–20 times higher costs than patients with drug-susceptible TB, due to relocation costs and longer pre-diagnosis and treatment periods involving more visits and procedures and inability to work [3, 4]. Patients who cannot afford to start or continue treatment will suffer from more extensive morbidity [5]. This may result in higher health system costs, and is likely to result in continued transmission [6].

Policy makers need to understand patient costs to assess how many families face catastrophic costs, to identify the main cost components in TB diagnosis and treatment that lead to catastrophic costs, to develop mitigation policies and to identify and tackle bottlenecks in access to and continuation of TB and MDR-TB treatment. Thus, measurement of financial burden and the main cost drivers for TB and MDR-TB diagnosis and treatment is needed. We conducted such a survey in three different settings; in Ethiopia, Indonesia, and Kazakhstan. We adapted a previously developed tool to estimate TB patients’ costs that has been implemented in several countries. That work had a positive impact resulting in improvements in access, nutrition support, adoption of a shorter treatment regimes, and the inclusion of TB services under insurance [7–9]. However, this tool was not meant to include both TB and MDR-TB patients and compare costs between both patient groups. The tool was, therefore, adapted for inclusion of MDR-TB patients’ costs to determine the main cost drivers for TB and MDR-TB diagnosis and treatment. The results observed in the three study countries were presented and discussed in in-country workshops for policy makers, focusing on ways to relieve the financial burden of diagnosis and treatment for TB and MDR-TB patients. The results from the surveys in Ethiopia, Indonesia, and Kazakhstan are described here together with identified mitigation strategies.

## Methods

### Study design

We conducted a cross-sectional survey in six public hospitals (and their satellite clinics) providing TB and MDR-TB (from now on referred to as (MDR)TB) services in Ethiopia, Indonesia, and Kazakhstan. These three countries were selected purposefully as to have representation from three different settings: one in Africa, one in South-Asia and one in Central Asia. Details on methods and results per country are available in the individual country reports and a summary report [10–13].

The (MDR)TB patients were interviewed once, at the health facility. In Ethiopia patients were interviewed at all three MDR-TB hospitals (St. Peters and ALERT in Addis Ababa and University of Gondar Hospital in Gondar). In Indonesia patients were interviewed at two MDR-TB referral hospitals on Java Island (Persahabatan hospital in Jakarta and Dr Moewardi hospital in Solo) and five satellite sites. In Kazakhstan patients were interviewed at one MDR-TB hospital caring for MDR-TB patients from Akmola oblast and its satellite sites providing directly observed therapy (DOT) for (MDR)TB patients in Kokshetau city.

The previous version of the questionnaire [7] was used as the basis for a new generic questionnaire. It was shortened to exclude questions not informative with respect to TB costs (on delays in health seeking behavior, on additional costs for other illnesses, and on impact of disease on social life). Included were some questions expected to be applicable mostly for MDR-TB patients; on adverse effects of treatment and related costs, relocation costs, and on receiving incentives and enablers (e.g. transport or food vouchers).

We did not aim to collect longitudinal data of patients covering the full pathway of diagnosis and treatment, since this would make data collection a lengthy and complicated undertaking when done prospectively. Retrospective data collection over a prolonged period of time would yield unreliable results [9], especially for MDR-TB patients, probably leading to underestimation of costs. To get insight in costs of the different phases of diagnosis and treatment of (MDR) TB, we included patients in different phases of treatment.

### Study population

We categorized and selected patients from five groups of TB and MDR-TB patients, representing different phases of diagnosis and treatment:

1. TB patients who completed at least 1 month of treatment and were within last month of the intensive phase of drug-susceptible TB treatment;
2. TB patients who started at least 3 months previously with the continuation phase of drug-susceptible TB treatment;
3. Patients diagnosed with MDR-TB within the month before the interview;
4. MDR-TB patients who started at least 3 months previously with the intensive phase of MDR-TB treatment;
5. MDR-TB patients who started at least 3 months previously with the continuation phase of MDR-TB treatment.

We excluded patients not consenting to the study, those not able to answer the questions in the interview, and those younger than 21 years of age since most of those below the age of 21 are not economically independent and still mainly live on their parent’s earnings. Also, we excluded patients who died or transferred out while on treatment because of logistic difficulties of reaching them or family members for reliable information. In Indonesia, bedridden patients were also excluded as these could not be interviewed in a private environment. In Kazakhstan, two additional exclusion criteria were applied: 1. patients diagnosed by Xpert MTB/RIF were excluded as this diagnostic tool only very recently had been introduced and only small numbers of patients had been diagnosed with it, and 2. patients who receive home-based care, as they are a small group with very distinct costs compared to other patients.

### Sampling

We aimed to include 50 patients per group in each of the three countries. We applied consecutive sampling, inviting all patients coming to the included health facilities to participate in the study until the target sample size was reached or until the end of the study period, whichever came first.

### Data collection

Structured interviews were conducted by trained interviewers with (MDR)TB patients in different stages of treatment. Eligible patients were invited to participate in the interview by the doctor or nurse they were seeing during their scheduled visit to the health care facility. After this visit, those patients wishing to participate in the study were sent to a separate room where they were interviewed by the study staff, i.e. not involved in the patients’ care. Before the start of the interview, written informed consent was obtained. Through a structured questionnaire we collected data on costs related to the diagnosis and treatment of (MDR)TB patients, as well as background information of the patients (age, sex, treatment type and phase, socioeconomic status, ethnicity and distance to health facilities). To minimize recall bias [9], we restricted collection of most cost data to the last 3 months; but major coping costs were not restricted to this period.

In each country, the structured questionnaire was translated from English to the local language, adapted to the local context for some questions (e.g. insurance types, type of health care facility, reimbursement schemes), and translated back into English by another individual to check for translation and interpretation errors. The questionnaire was pretested to check for clarity on 3–5 patients per country before it was finalized. Face-to-face interviews were conducted in March 2013 (Ethiopia), February-March 2013 (Indonesia), and September-October 2012 (Kazakhstan) at the selected health care facilities. The questionnaire included cross-checks and the interviewers were trained to double-check unusually high costs when reported by the patients. Data on costs were collected in the local currency.

### Data analysis

For each country, data were entered in a separate pre-designed data entry file (Microsoft Excel for Ethiopia; EpiData (www.epidata.dk) for Indonesia and Kazakhstan) and analyzed (Microsoft Excel for Ethiopia; STATA/SE 11.1 for Windows (Stata Corp., College Station, Texas, USA) for Indonesia, SPSS v20 IBM, New York, USA) for Kazakhstan).

We calculated costs of getting a (MDR)TB diagnosis, costs of treatment (in the intensive and continuation phase of (MDR)TB treatment) and financial values involved in coping as explained below and summarized in Table 1.

**Table 1:** Methods used to estimate different types of costs for TB diagnosis and treatment

Type of cost	Elements included in cost type	Methods used to calculate costs
Diagnostic (for those in intensive phase)	Food, travel, accommodation, medical costs, and loss of income during visits	Summed direct and indirect costs of visits
		Indirect costs (income loss) as calculated from total time spent x income/time
Treatment (excluding for those just diagnosed with MDR-TB)	DOT and drug collection visits, follow-up tests, food, travel, treatment of adverse events^a^, supplements^b^, hospitalization^c^, and loss of income	Summed direct and indirect costs, multiplied by number visits/week, weeks/ month, and internationally defined duration of treatment phase
		Indirect costs (income loss) for DOT as calculated from total time spent x income/time
Other Costs	Direct and indirect costs of accompanying persons/attendants	Summed costs related to diagnosis or treatment visits
Coping strategies	Amount borrowed, assets sold	Summed costs

^a^Assuming that all costs for these elements had been made before the time of the interview (hence, costs were not extrapolated to the treatment phase)

^b^Summed direct costs over last month *x* internationally defined duration of treatment phase

^c^In Ethiopia and Indonesia: costs reported up until time of interview. For Kazakhstan, summed direct costs over last month *x* internationally defined duration of treatment phase; summed indirect costs (income loss) for hospitalization as calculated based on internationally defined duration of intensive phase *x* income/time

#### Costs for (MDR) TB diagnosis

Costs were obtained per diagnostic visit. Direct costs included all out-of-pocket payments that the patient had to make, such as paying administration fees, paying for laboratory tests, X-ray, and drugs, for food and accommodation, and for transportation to and from the hospital. Direct costs were summed up per cost item over all visits, after which the sums of the cost items were summed up in a total of direct costs per patient. Indirect costs (loss of income) were calculated by multiplying the total number of minutes spent on diagnostic visits with the patient’s income per minute before diagnosis of TB.

#### Costs for (MDR) TB treatment

Cost items for (MDR) TB treatment included costs made because of taking or picking up drugs at the clinic, costs for follow-up tests, supplements, hospitalization, and treatment of adverse events. Costs for taking or picking up drugs were reported for a typical visit to take or pick up drugs. To get the total costs per month, individual cost items per visit were summed up and the total costs per month were calculated by multiplying these costs with the number of times per week that drugs were taken/picked up and the number of weeks per month (4.3). Indirect costs were calculated by multiplying the turn-around-time in minutes for a typical visit with the number of times per week that drugs were taken/picked up, the patients’ income per minute, and 4.3 weeks per month. These monthly costs were subsequently extrapolated over the complete treatment phase using the internationally defined durations of the different treatment phases: 2 months of intensive phase and 4 months of continuation phase for new TB patients, 3 and 5 months for retreatment patients and 8 and 12 months for MDR-TB patients [14, 15]. If patient had been longer in their treatment phase at the time of the interview, we assumed they were in the last month of the respective phase during the interview. The main outcomes therefore were total costs incurred by the patient during the phase (intensive or continuation) of treatment they were in.

Costs for follow-up tests were reported from the start of TB treatment till the interview. Since it was assumed that in a typical TB treatment phase, only one or two follow-up tests would be needed, no extrapolation was applied to obtain the costs per treatment phase for patients being treated with TB regimens. To calculate the costs per treatment phase for MDR TB patients, the total costs were multiplied by the internationally defined duration of the treatment phase of the patient, divided by the number of months that the patient had been in that treatment phase.

Costs for supplements were reported over the past month. To obtain the total cost per month, individual cost items were summed up and extrapolated to the total treatment phase. We considered adverse events needing treatment unlikely to occur and did not apply extrapolation of the costs reported to the complete treatment phase.

In Ethiopia and Indonesia most TB and MDR-TB patients are not hospitalized, unless cases are severe or experience serious side effects from treatment. In these two countries we therefore assumed that hospitalization did not occur after the interview and we did not extrapolate the costs of hospitalization to the complete treatment phase. In Kazakhstan however, most patients are hospitalized during the full intensive phase of treatment. As patients are not able to work when hospitalized, loss of income in Kazakhstan was calculated assuming hospitalization for the duration of the intensive phase.

#### Coping costs

Coping with the financial impact of TB treatment involves multiple strategies, such as borrowing money, asking for donations from family and friends, using savings, selling assets costs and cutting down other expenses. We asked patients for the financial impact of their disease on their family and the coping strategies used. Costs were defined as loss of household income after TB diagnosis (indirect costs), amounts borrowed, and market value of assets sold (both defined as direct costs). We did not extrapolate any of these costs since reduction in household income was reported as monthly reduction in income and it remained unknown when the income had changed. Besides, we assumed that borrowing money and selling assets were one-off actions.

Since the distributions of almost all costs were highly skewed towards higher values, we chose to present median values with 25th and 75th percentiles (also called the interquartile range (IQR)). The total financial value for coping strategies reported by the patient was calculated.

We converted all costs into US Dollar using the average daily midpoint exchange rate over the data collection period [16]. Over this period, the average exchange rates for 1 USD were 18.60 Ethiopian Birr, 9689.86 Indonesian Rupiah, and 148.35 Kazakh Tenge.

## Results

In total 197 TB patients and 406 MDR-TB patients participated in the three countries: 25 TB patients and 169 MDR-TB patients in Ethiopia; 118 TB patients and 143 MDR-TB patients in Indonesia; plus 54 TB patients and 94 MDR-TB patients in Kazakhstan (Table 2). In Ethiopia, the time period allocated for data collection turned out to be too short and it was decided to focus on reaching the targets for the number of MDR-TB patients. In Kazakhstan, the number of eligible TB patients treated at the selected healthcare facilities was below 50 during the period of data collection. In all three countries, the majority of patients were pulmonary sputum smear positive patients.

**Table 2:** Patient characteristics

	Ethiopia		Indonesia		Kazakhstan	
	*n*	(%)	*n*	(%)	*n*	(%)
Patient group						
Intensive phase of standard (re)treatment regimen	12	(6.2)	62	(23.8)	41	(27.3)
Continuation phase of standard (re)treatment regimen	13	(6.7)	56	(21.5)	13	(8.7)
Just diagnosed with MDR-TB	21	(10.8)	29	(11.1)	2	(1.3)
Intensive phase of MDR-TB treatment	85	(43.8)	55	(21.1)	62	(41.3)
Continuation phase of MDR-TB treatment	63	(32.5)	59	(22.6)	32	(21.3)
Type of TB						
Pulmonary smear positive	176	(91.2)	166	(63.6)	121	(80.7)
Pulmonary smear negative	4	(2.1)	72	(27.6)	27	(18.0)
Extrapulmonary	13	(6.7)	16	(6.1)	2	(1.3)
No information	1	(0.5)	7	(2.7)	0	(0.0)
Gender						
Male	107	(55.2)	138	(52.9)	100	(66.7)
Female	87	(44.8)	120	(46.0)	50	(33.3)
No information			3	(1.2)		
Age (years)						
21–29	110	(56.7)	62	(23.8)	47	(31.3)
30–39	49	(25.3)	71	(27.2)	43	(28.7)
40–49	20	(10.3)	66	(25.3)	42	(28.0)
50+	15	(7.7)	61	(23.4)	18	(12.0)
No information			1	(0.4)		
HIV						
Positive	41	(21.1)	8	(3.1)	0	(0.0)
Negative	146	(75.3)	128	(49.0)	150	(100)
not tested/unknown	7	(3.6)	125	(47.9)	0	(0.0)

The median (IQR) number of visits needed for a TB diagnosis was three (2–5) in Ethiopia, three (2–4) in Indonesia, and two (2–3) in Kazakhstan. For Ethiopia, the number of respondents on TB diagnosis was small, and four out of five were from Gondar with a large and remote catchment area. The median time spent per visit for those patients was 43 h for a total median time spent for diagnostic visits of 144 h. The median (IQR) total time in minutes needed for diagnostic visits was 355 (130–600) in Indonesia and 120 (78–273) in Kazakhstan.

### TB illness related costs

The median costs (with IQR) for patients in the three countries are shown in Table 3. Costs are separated for diagnostic and treatment expenditure. Also, we show direct (out of pocket) and indirect (foregone income) costs separately. The median estimated total costs for diagnosis and treatment during the current TB treatment episode was USD 260 in Ethiopia, USD 169 in Indonesia, and USD 929 in Kazakhstan, respectively. The median estimated costs for MDR-TB patients were 7.1, 13.9 and 3.4 times higher: USD 1838 in Ethiopia, USD 2342 in Indonesia, and USD 3125 in Kazakhstan, respectively.

**Table 3:** Summary table on median costs (interquartile ranges) in US dollars for TB and MDR-TB patients in the three study countries, related to costs for diagnosis, and treatment in the intensive phase and continuation phase

	TB			MDR-TB		
	Ethiopia	Indonesia	Kazakhstan	Ethiopia	Indonesia	Kazakhstan
Direct pre(diagnosis) costs (costs in last 3 months)	14 (4–109)	33 (9–64)	5 (1–13)	68 (35–191)	39 (12–63)	N.A.^b^
Indirect pre(diagnosis) costs (costs in last 3 months)	0 (0–30)	4 (0–9)	3 (1–5)	0 (0–8)	3 (1–6)	N.A.^b^
Total pre(diagnosis) costs (costs in last 3 months)	14 (6–129)	35 (16–69)	9 (4–19)	75 (40–191)	46 (16–82)	N.A.^b^
Direct treatment costs						
Subtotal for intensive phase	104 (10–231)	41 (8–108)	0 (0–74)	639 (259–968)	596 (342–1035)	165 (0–541)
Subtotal for continuation phase	80 (34–156)	59 (17–224)	179 (90–328)	634 (458–1048)	976 (558–1584)	754 (344–2022)
Indirect treatment costs						
Intensive phase	0 (0–34)	10 (0–40)	404 (303–674)	220 (89–374)	315 (153–848)	1537 (0–2696)
Continuation phase	0 (0–4)	9 (0–57)	104 (70–159)	73 (1–375)	254 (0–504)	227 (0–300)
Total treatment costs						
Intensive phase	119 (19–260)	52 (17–134)	607 (317–809)	831 (462–1525)	1079 (600–2299)	1914 (175–3370)
Continuation phase	128 (34–177)	82 (26–286)	319 (236–702)	931 (494–1296	1227 (730–1846)	1202 (657–2245)
Total (pre)diagnosis and treatment costs^a^	260	169	929	1838	2342	3125

^a^Sums are based on adding up medians from different groups of patients, and therefore must be interpreted with caution

^b^Not available as only two patients were interviewed with a diagnosis of MDR-TB in the last month

Treatment costs were much higher than diagnostic costs in all countries, both for TB and for MDR-TB patients, with median diagnostic costs ranging between USD 9 and USD 75 (Table 3). In Ethiopia and Indonesia but not in Kazakhstan, direct costs for treatment where higher than indirect costs related to treatment. In Kazakhstan, estimated indirect costs were high because of hospitalization in the intensive phase.

The main cost components related to (MDR) TB diagnosis and treatment varied between countries.

In Ethiopia the highest cost element in the diagnostic phase was for food expenditure and for food supplements during treatment, both for TB and MDR-TB patients. In Indonesia the largest cost share during diagnosis was for travel and food for TB patients, and for laboratory tests and administration fees for MDR-TB patients. For both TB and MDR-TB patients, travel expenditure was the highest cost element during treatment. In Kazakhstan, transport expenditure was responsible for most costs during diagnosis, and indirect costs of hospitalization and direct costs related to food supplements and travel for DOT visits during treatment.

### Socio-economic impact of TB illness related costs

Table 4 shows the main indicators of the socioeconomic impact of MDR-TB disease in the three countries. Most patients reported income loss due to TB illness, ranging from 33 % of TB patients in Ethiopia to 100 % for MDR-TB patients in Kazakhstan (where no outpatient treatment during the intensive phase was available at the time of the data collection). The median value of this reduction in income was 100 % except for TB patients in Indonesia 25 %). A highly varying proportion of patients received assistance, ranging from 17 % of TB patients in Kazakhstan to 73 % of MDR-TB patients in Ethiopia. However, in all countries the amount of financial assistance received in general was low, including through health insurance. The proportion of patients who sold property or took out loans to cope with TB related costs, was especially high in Ethiopia: 56 % of TB patients and 41 % of MDR-TB patients took out loans.

**Table 4:** The main indicators of financial impact of TB illness experienced by the (MDR) TB patients in the three countries

	Ethiopia		Indonesia		Kazakhstan	
	TB	MDR-TB	TB	MDR-TB	TB	MDR-TB
Patients who were primary income earner before TB illness	N.A.^b^	N.A.^b^	44 %	24 %	61 %	53 %
Patients who lost their job	76 %	72 %	26 %	53 %	31 %	41 %
% of patients reporting income loss due to TB	92 %	79 %	38 %	70 %	67 %	56 %
% reduction in median income *(for those reporting an income change)*	100 %	100 %	25 %	100 %	100 %	100 %
Patients hospitalized for TB	36 %	82 %	33 %	62 %	98 %	100 %
median duration of hospitalization (days)^a^	40	80	7.5	10	90	195
Patients who received assistance from government or other organizations	24 %	73 %	22 %	34 %	17 %	27 %
median value of assistance in last 3 months (USD)^c^	76	33	0	41	88	31
Coping costs						
patients who sold property	24 %	38 %	3 %	21 %	0 %	1 %
patients who took out loans	56 %	41 %	9 %	27 %	0 %	4 %
patients who received donations from family/friends	N.A.	N.A.	32 %	43 %	57 %	66 %
Patients with health insurance	0 %	1 %	22 %	25 %	0 %	1 %
Of those, patients who received reimbursements	0 %	0 %	N.A.^d^	N.A.^d^	0 %	0 %

^a^For those patients in hospitalized at time of interview, assuming hospitalization for patients during standard duration of intensive phase

^b^Not available as this question was taken out of the locally used questionnaire

^c^For Ethiopia and Kazakhstan, this includes the value of vouchers; for Indonesia it only includes cash assistance

^d^In principle, insured patients receive specified services for free. However, not all services provided are necessarily included

**Fig. 1:**
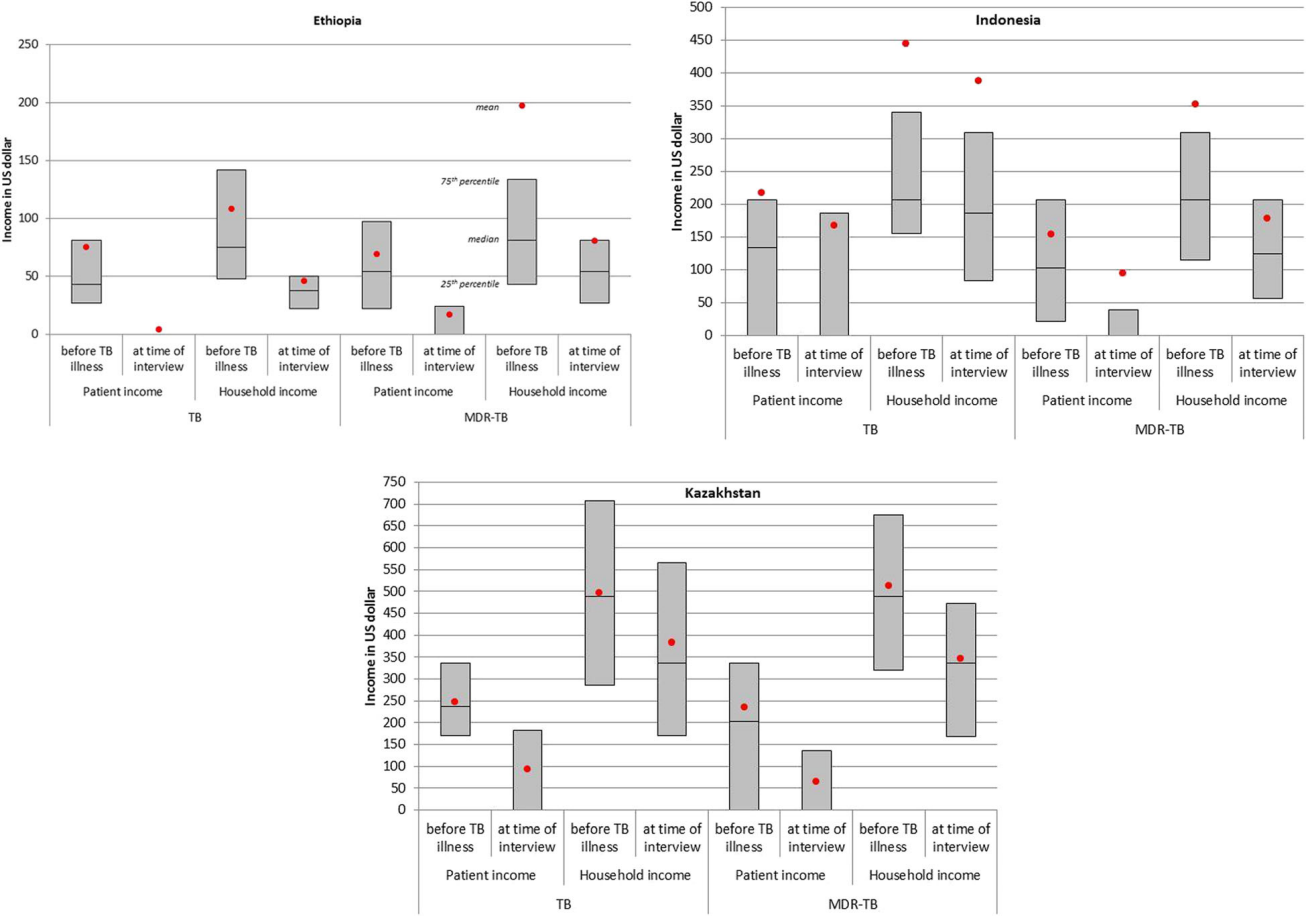
Box plots showing mean, median and interquartile range of patient and household income before TB illness and at the time of the interview, stratified for TB and MDR-TB patients. Plots are provided separately for patients interviewed in Ethiopia, Indonesia and Kazakhstan. Note the different y-axis scales used. Whiskers are not included as distributions are highly skewed to high incomes, with some patients and household having an income far above the 75th percentile

Figure 1 shows patient and household income before TB illness and at the time of interview. Mean incomes were much higher than median incomes, especially in Indonesia and to a lesser extent in Ethiopia, representing the highly skewed distributions with a few patients have relatively much higher incomes than the rest.

In Ethiopia the median TB and MDR-TB patient income fell from USD 43 and USD 54 to before TB illness, respectively, to zero at the time of the interview. The fast majority (88 % of TB patients and 76 % of MDR-TB patients) did not have any income after (MDR) TB diagnosis, compared to 8 and 14 % before (MDR)TB diagnosis. The median monthly household income of TB patients dropped by 50 % (from USD 75 to USD 38), and by 33 % (from USD 81 to USD 54, respectively). Although many patients were primary income earners before TB diagnosis, household members started to work more to compensate for lost income. The total costs of TB and MDR-TB diagnosis and treatment equaled 4.6 and 24.9 months of pre-diagnosis household income.

In Indonesia, the median TB and MDR-TB patient income dropped from 134 and 103, respectively, to zero. The proportion of TB patients with no formal income increased from 29 % before diagnosis to 52 % at the time of the interview, and from 22 to 74 % for MDR-TB patients. The median household income dropped by 10 % (from USD 206 to 186) and 40 % (from USD 206 to 124), respectively. The total costs of TB and MDR-TB diagnosis and treatment equaled 0.82 and 11.4 months of pre-diagnosis household income.

In Kazakhstan, the median TB and MDR-TB patient income dropped from USD 236 and 202 USD to zero, respectively. Fifty-nine percent and 67 % of TB and MDR-TB patients, respectively, did not have any income at the time of interview, compared to 13 and 36 % before diagnosis. The median household income of TB and MDR-TB patients dropped by 20 % (from 708 to 566 USD), and 31 % (from 489 to 337 USD), respectively. As in Ethiopia, many patients were primary income earners before TB diagnosis, and household members started to work more to compensate for lost income. In Kazakhstan, the median household income dropped by 31 % both among TB and MDR-TB patients, and the total costs of TB and MDR-TB treatment equaled 2.8 and 9.3 months of median pre-diagnosis household income.

### Mitigation policy options

Policy options for mitigating patient costs due to (MDR) TB were listed during national workshops with participants representing different Ministries, Universities, hospitals, non-governmental organizations (NGOs), civil society organizations (CSOs), and patients. Options related to TB service improvements prioritized in all three countries were 1) to ensure that the policy of free care for all (MDR) TB services is fully implemented and 2) that services are brought closer to patients, followed by social service improvements related to 3) inclusion of direct (transport, food support) costs in social support schemes provided through TB services, 4) inclusion of indirect (sick leave allowance) costs in social protection schemes, and 5) improvements of employment protection. Note that these recommendations are not mutually exclusive – to improve the situation of especially MDR-TB patients, it may be necessary to apply more than one strategy at the same time.

## Discussion

The findings from all three countries showed that, although MDR-TB diagnosis and treatment services are supposed to be free for patients, patients have other direct and indirect costs and the financial impact was significant for most patients. For most respondents, direct and indirect costs increased while income decreased. The estimated costs of MDR-TB patient diagnosis and treatment were 3.4–13.9 times greater than those for other TB patients, mainly due to the longer time period for treatment. Aggravating this situation, MDR-TB patients more often lost their jobs.

We probably underestimated direct and indirect costs in our study. Firstly, costs for the pre-diagnosis period may have been underestimated as patients may spend a long time getting an accurate diagnosis, making full recall difficult. Secondly, for some patients treatment duration may be prolonged, e.g. due to missed doses during TB treatment or lack of culture conversion during the intensive phase of MDR-TB treatment. Thirdly, we only included costs of the current treatment episode while especially MDR-TB patients may have been treated previously. Fourthly, indirect costs presented here do not include costs after the end of treatment, especially further loss of income for those who have lost their jobs or who have developed disabilities not allowing them to do the work they did before. Fifthly, loss of income was estimated only as a result of time spent obtaining diagnosis and for getting treatment. In reality, some patients may not work at all because they are not feeling well, because they lost their job, or because they are not allowed to work (i.e. in Kazakhstan). This may be the reason why we found a smaller proportion of costs incurred before TB diagnosis than the 50 % estimated in a recent systematic review [2]. That is why the updated version of the questionnaire –currently applied in several countries under leadership of WHO- also collects information on time off work. Of note, we did not discount financial assistance that patients had received. Although a substantial proportion of patients did report to receive financial assistance from the government or other organizations, the majority of patients received only incident and little to no actual reimbursements. So this would far from compensate patients’ actual costs including reduced income.

This study has several other limitations. Most importantly, due to limitations in time and budget, only patients being under care at health facilities were interviewed. It was not feasible to conduct interviews to collect data from people who did not attend a facility during the period of the study. Such people may have been too poor to seek diagnosis and treatment. Among those who initiated treatment, some stopped treatment – an unknown proportion because of associated costs - or died during treatment – the impact on family income would be greatest for those households. Therefore, the study population may have been biased against the less socio-economically vulnerable groups [17]. Globally, 16 % of MDR TB patients are lost to follow-up and another 16 % die during treatment [18]. Their families lose the income of the deceased household member. A substantial but unknown proportion of patients die before accessing appropriate diagnosis and treatment.

A consequence of our study design is that we did not collect total costs of (MDR) TB treatment per patient – which would have required longitudinal follow-up - but instead extrapolated costs per stage and to the total (MDR) TB episode. Also, the study was limited to a few public health facilities in Indonesia and Kazakhstan – all three MDR-TB treatment centers in Ethiopia were included - and thus, rather than providing an estimate of the costs incurred by the average (MDR) TB patient in those countries, it does give insight into the major cost components and it provides an idea of the financial burden that a free public health program poses on its patients.

Although many patients were primary income earners before TB diagnosis in Indonesia and Kazakhstan (results not available for Ethiopia), household members started to work more to compensate for lost income. Less MDR-TB than TB patients were primary income earners and on average they earned less than TB patients; this may be explained by the fact that most already were being treated for TB at the time of MDR diagnosis.

Transport costs to reach the DOT facility may be small, but may add up to a substantial amount if made every day during ambulatory treatment. For some patients, these costs can be brought down by bringing DOT facilities closer to the patients’ homes. It is important that the facility staff or community health workers do have sufficient expertise to manage MDR-TB patients, including those needed to recognize treatment failure and adverse drug reactions at an early stage to ensure patients can access clinical services when necessary and will not stop treatment [16]. Several reviews concluded that ambulatory and community-based MDR-TB models of care are equally or more effective than hospital-based models in treatment outcomes and may be more cost-effective 19–23]. However, even community-based treatment models may face high proportions of patients lost to follow-up [24] and economic support may still be required [25].

Only a few studies collected patient cost data specifically both for TB and MDR-TB patients and numbers of patients usually were small [2]. In Ecuador, average patient costs were estimated at USD 960 among 104 TB patients compared to USD 6880 for 14 MDR-TB patients [4]. In Cambodia, total household costs for eight MDR-TB patients was USD 1525 compared to USD 477 for 261 HIV-negative TB patients and USD 555 for eight HIV-positive TB patients [26]. Only in Brazil, patient costs were not very different for MDR-TB patients, although health service costs were 37 times higher: total household costs were estimated to be USD 266 for new TB patients compared to USD 333 for MDR-TB patients [27]. In the Dominican Republic, 20 out of 198 TB patients had MDR-TB. Total costs were estimated at UDS 3557 for MDR-TB patients compared to USD 908 for new patients [8]. Our study confirmed previous findings that in general MDR-TB patients face much higher costs than other TB patients as a result of longer duration of treatment, more adverse drug reactions due to the more toxic drugs used in MDR-TB treatment, and related need for (additional) hospitalization.

### Policy implications

The recommendations we made were similar to the ones based on studies with the previous version of the questionnaire, not specifically including MDR-TB patients [7]: bringing services closer to patients, reducing expenditures on transport and invested time, increasing efforts to find cases early to reduce indirect costs related to inability to work, informing health care workers and the public about TB diagnosis and treatment to reduce costs unrelated to TB, and including TB-related out-patient costs in social protection schemes (Table 5 and Table 6 in Appendix). Indonesia is rapidly expanding the number of satellite sites. All three countries are moving towards outpatient care, with expansion of DOT services in primary health care services. This study shows the importance of using freed up resources from hospital-based care to support patients during treatment.

**Table 5:** Summary of policy options to mitigate (MDR) TB patients’ costs considered per country

	Ethiopia	Indonesia	Kazakhstan
*TB service improvements*			
Ensure that policy of free care for all (MDR) TB services is fully implemented	X	X	X
Bring services closer to patients	X	X	X
Detect and treat MDR-TB cases earlier	X	X	X
Raise the awareness of health workers	X	X	X
Involve local NGO’s and civil society organizations		X	X
Reduce hospitalization			X
No unnecessary or substandard tests		X	
Obligatory treatment for MDR-TB patients		X	
*Social protection improvements*			
Include direct (transport, food support) costs in social support schemes provided through TB services	X	X	X
Include indirect (sick leave allowance) costs in social protection schemes	X	X	X
Improve employment protection	X	X	X
Reduce stigma and acceptance of outpatient treatment	X	X	X
Increase re-socialization and employment possibilities	X	X	X
Use social health insurance	X	X	
Consistency across social assistance programs and over time	X		
Assure continuation of education			X
Involve local NGO’s and civil society organizations		X	
Provide convenient lodging		X	
Empower patient groups that can support MDR-TB patients		X	

Based on results from the previous version of the tool, several countries took action to implement one or more of the identified solutions for TB patients [7]. For example, policy makers in Ghana agreed to include TB care interventions as part of its pro-poor strategies in the delivery of health care and nutrition guidelines were developed to address the specific needs of TB patients. Given the identified high burden for female TB patients in Ghana, the national tuberculosis program (NTP) focused on addressing gender-sensitive challenges of poor TB patients. Also the insurance coverage for all TB patients was increased to also cover health-related costs other than anti-tuberculosis treatment. In Vietnam, the NTP decided to increase the involvement of the private sector in public-private-mix projects focusing on reducing travel, accommodation and hospitalization costs for TB patients and guardians. Also, the NTP worked on the expansion of its NTP network to provide TB services at more public and private hospitals. In the Dominican Republic the Ministry of Health decided to move forward with allocating public funds for food supplements for TB patients and including in- and outpatient TB services in the national health insurance schemes. In Kenya, TB treatment services were decentralized, local partners were approached for sputum sample transport reduce patients’ transport costs and time spent on the road, and other health programs were approached for nutritional support of TB patients. A TB and poverty sub-committee was convened to develop a comprehensive pro-poor approach within the routine TB program [9]. This shows that action may be taken only after studies can show policy makers what the issues are.

Both in Ethiopia and Indonesia, a considerable proportion of MDR-TB patients may not start treatment after diagnosis and another considerable proportion is lost to follow-up before completion of treatment. We do not know in how far economic consequences are a key reason for this but they may be a relevant contributor. In Ethiopia as many as 29 % of patients diagnosed with MDR-TB may not have started second-line drug treatment and 3 % are lost to follow-up during treatment (unpublished data: Ministry of Health progress report to the Green Light Committee, April 2013). In Indonesia around one-third of diagnosed MDR-TB patients is not started on MDR-TB treatment, whereas up to one-third of those starting treatment is lost to follow-up during treatment (unpublished NTP data, Indonesia).

Treatment cost data were collected during a single interview and extrapolated over the treatment phase the patient was in during the interview, i.e. intensive or continuation phase. As costs were estimated per treatment phase and not per patient, it means that this study did not yield total costs of (MDR) TB treatment incurred per patient. To give an idea of the costs of a total episode of (MDR) TB, we did add median costs per stage, thus assuming that patients interviewed per stage were representative of all patients. These summed medians must therefore be interpreted as crude estimates, meant to indicate what were the main cost drivers. With this cross-sectional method we were able to capture the major cost components in a relatively short timeframe. Capturing the total costs per patient requires follow-up of a sample of patients during their treatment, which may take more than 2 years for MDR-TB patients and takes at least 6 months for TB patients. To get an exact estimate of total costs incurred, other methods than (repeated) interviews would have been required, such as patient diaries. However, it is known that it is difficult to motivate patients to keep diaries for a longer time period and this may lead to selective dropout of the less well educated and socially engaged patients.

## Conclusions

In conclusion, while the financial burden of MDR-TB patients was (much) higher than that of TB patients in all three countries, all patients experienced substantial socioeconomic impact of TB disease, most importantly due to inability to work and job loss. If the patient is the breadwinner of the family, the combination of lost income and extra costs generally is catastrophic. A too high financial burden may cause patients to not get diagnosed, to not start treatment, or to stop treatment, leading to prolonged transmission of the disease to others. Patients stopping treatment as soon as they feel better may need retreatment, which is more expensive, takes longer and is more toxic than initial treatment. Therefore, it should be a priority of governments to relieve the financial burden especially for MDR-TB patients. The cost mitigation options in all three countries should be used to prepare an action plan for mitigating patient costs under the guidance of NTP, indicating main stakeholders, and with whom, how and when the option can be worked out into a strategy, and when and how this strategy can be implemented. However, the effectiveness of such strategies will depend on the countries’ willingness and ability to address these problems.

## Appendix

**Table 6:** Policy options to mitigate (MDR)TB patients’ costs considered per country (expansion of Table 5 in manuscript)

	Ethiopia	Indonesia	Kazakhstan
*TB service improvements*			
Ensure that policy of free care for all (MDR) TB services is fully implemented. Agreements need to be in place so that presumed TB patients can make use of the necessary diagnostic tools for free.	X	X	X
Bring services closer to patients. Further decentralization should reduce patient expenditures on transport and patient time and should reduce detection and treatment delays, especially for MDR-TB patients. For areas where there is no public transport, transport for patients or home visits should be arranged. This includes improving downward referral from national or provincial MDR-TB treatment centers to local community health centers.	X	X	X
Detect and treat MDR-TB cases earlier. Especially detection of drug-resistant TB should reduce the time to appropriate treatment, and thus reduce direct and indirect treatment costs for patients, especially the amount of income lost due to inability to work during initial first-line drug treatment. Full implementation of new diagnostics such as Xpert MTB/RIF should reduce time to diagnosis and thus patient costs.	X	X	X
Raise the awareness of health workers. Provide education and training of primary level health workers to recognize suspects and ensure speedy diagnosis, and to follow up on cases and contact tracing.	X	X	X
Involve local NGO’s and civil society organizations to support patients and hereby improve (MDR) TB treatment adherence.		X	X
Reduce hospitalization. Kazakhstan has moved in recent years from full in-patient treatment to partial outpatient treatment, usually in the continuation phase. The country plans to move towards full outpatient care. This has the potential to greatly reduce indirect patient costs.			X
No unnecessary or substandard tests. Sometimes, tests are being prescribed by physicians that are not needed (e.g., X-ray for diagnosis of smear-positive TB patients). Private laboratories sometimes use substandard tests (e.g., IS*6110* based PCR for detection of *Mycobacterium tuberculosis*) and serological tests. Such tests are not only unnecessary, but also may importantly increase the costs of (MDR) TB diagnosis.		X	
Obligatory treatment for MDR-TB patients may be needed in parts of the country where a large proportion of MDR-TB patients refuses MDR-TB treatment, due to lack of knowledge or support, to protect the community against the spread of MDR-TB. MDR-TB patients may fear the costs and side effects related to MDR-TB treatment. Patient education, installation of patient organizations (as is starting up now in different hospitals), and provision of living allowances may help to remove some of these obstacles.		X	
*Social protection improvements*			
Include direct (transport, food support) costs in social support schemes provided through TB services. Such incentives and enablers should reduce direct costs associated with TB treatment and improve treatment adherence.	X	X	X
Include indirect (sick leave allowance) costs in social protection schemes. Review, standardize and expand current social protection mechanisms and schemes by the government. Social protection schemes, including temporary disability allowances, should be made available to those (MDR) TB patients who need it, from the moment they are diagnosed. Include social protection for (MDR) TB under disability policy strategies while ensuring that the protection is provided from the time of confirmed diagnosis to those who are at risk of becoming poor or not seeking or completing treatment. Professional guidance by health care workers or social workers for submitting applications for social support is needed for many patients. Possibilities for agreements on delaying or waiving payments (e.g. mortgage loans, school fees) are to be investigated.	X	X	X
Improve employment protection. Advocate for regulations and policies that mandate that both public and private employers pay employees (a portion of) their salary while they are unable to work. Also advocate for patients to be able to return to previous positions once they are fully cured and clinically fit to perform their assignments.	X	X	X
Reduce stigma and acceptance of outpatient treatment. Improve education to the public on TB and MDR-TB, e.g. through primary level services, in order to reduce stigma of (MDR) TB and reduce fear of transmission during outpatient treatment.	X	X	X
Increase re-socialization and employment possibilities. Develop mechanisms to involve socially vulnerable patients in different re-socialization activities provided e.g. through temporary, assisted living facilities. Develop mechanisms to involve patients in income generating activities and advocate government to support this, for example through microfinance.	X	X	X
Use social health insurance. Advocate with government to incorporate TB services in the future social health insurance system to provide sustainable financing. Also advocate for social protection to be included in the benefits package on the grounds that this will reduce severity of illness and transmission and thus save on treatment costs.	X	X	
Consistency across social assistance programs and over time. The data collected on vouchers indicates that the amounts provided are very low compared with the patient costs and taking into account reductions in income. In addition there may be inconsistency in the amounts provided across facilities and over time. It is recommended that the government develops a standard.	X		
Assure continuation of education. When rendered non-infectious, children and students need to be able to continue their education.			X
Involve local NGO’s and civil society organizations and empower community health workers in provision of (MDR) TB drugs to improve (MDR) TB treatment adherence, since this will increase the population that can be targeted.		X	
Provide convenient lodging to those MDR-TB patients who cannot travel back and forth for receiving DOT. Since MDR-TB treatment roll out is still ongoing distances that MDR-TB patients have to travel for receiving DOT can be long in Indonesia and this may mean that patients need to move to a shelter close to the PMDT site. It is expected that the number of patients needing such housing will decrease with the roll out of the PMDT program.		X	
Empower patient groups that can support MDR-TB patients in a practical way during MDR-TB treatment. Being a new development in Indonesia, MDR-TB peer educator groups are being set up by ex MDR-TB patients. MDR-TB patient support groups provide information to MDR-TB patients regarding side effects, reimbursements systems, etc., and thus serve as a valuable and easily accessible information point to MDR-TB patients.		X	

## Abbreviations

AHRI: Armauer Hansen Research Institute
CSO: Civil society organization
DOT: Directly observed therapy (for (MDR)TB)
HIDN: Office of Health Infectious Disease and Nutrition
IQR: Interquartile range
MDR: Multi-drug resistance (i.e. resistance to rifampicin and isoniazid)
NGO: Non-governmental organizations
NTP: National Tuberculosis Program
TB: Tuberculosis
TORG: Tuberculosis Operational Research Group
USAID: United States Agency for International Development
USD: United States dollar
WHO: World Health Organization
